# Unlocking Efficient O_2_ Electroreduction in Conductive MOFs via Enhanced Mass Transport

**DOI:** 10.1021/acscentsci.2c00773

**Published:** 2022-07-08

**Authors:** Michael Stodolka, Jihye Park

**Affiliations:** Department of Chemistry, University of Colorado Boulder, Boulder, Colorado 80309, United States

Electrically conductive metal–organic frameworks (MOFs) have captured the curiosity of numerous chemical disciplines to harness their intrinsic properties for energy storage, electrochemical sensing, and electrocatalysis.^1,2^ While this excitement has led to a proliferation of reported conductive MOFs, many have yet to reach their full potential in an integrated device setting.^3^ In this issue of *ACS Central Science*, Dincă, Unwin, and co-workers utilize gas diffusion electrolysis and nanoscale electrochemical measurements to obtain the oxygen reduction reaction (ORR) with high current density in a Ni_3_(HITP)_2_-based electrode.^1^

As global demand for feedstock chemicals continues to increase rapidly, the development of new efficient electrocatalysts will be critical to maintaining adequate supplies of goods from plastics to fertilizers.^1,4^ Specifically, MOFs have been highly sought-after designer materials as their metal nodes and organic linkers may be independently tuned for the targeted application.^5^ Conductive MOFs are desirable for electrocatalysis over metal nanoparticles, sacrificial MOF-derived materials, or heterogeneous catalysts due to their intrinsically high internal surface area stemming from well-defined pores. Additionally, MOFs have some advantages over homogeneous electrocatalysts, where the catalyst is often too far from the electrode to be effective.^6^ Thus, MOFs’ accessible active sites and tunable pore architecture make them ideal candidates for electrocatalytic processes, such as the ORR. However, until now, conductive MOFs have shown much lower current densities (ranging from 0.5 to 0.8 mA cm^–2^) in electrocatalysis compared to currently available heterogeneous catalysts, such as metal nanoparticles that measure up to 200 mA cm^–2^.^2,7^

The common practice for determining the catalytic activity of porous materials involves drop-casting an ink of the material onto a rotating ring disk electrode (RRDE) and measuring the current density of the RRDE for the ORR in a two-compartment H-cell.^8^ Unfortunately, this method may convolute limitations of catalytic activity that are dependent on the mass transport of dissolved oxygen to catalytic sites and the intrinsic catalytic activity of the material.^6,7^ In most 2D MOFs, the issue of mass transport of oxygen to active sites may be exacerbated by the close stacking of layers. In their work “Thousand-Fold Increase in O_2_ Electroreduction Rates with Conductive MOFs”, the authors separate these two factors by mounting an isostructural family of conductive MOFs on a gas diffusion electrode (GDE) and regulating the flow of oxygen to the electrode.

**Figure 1:**
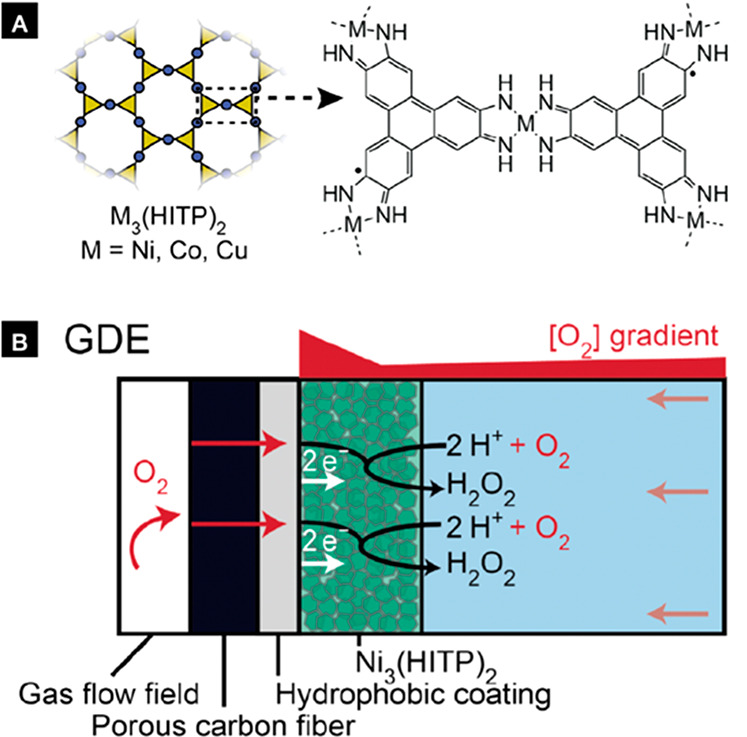
(a) Structure of the M_3_(HITP)_2_ motif. (b) Schematic for the gas diffusion electrode (GDE) for the ORR. Reproduced with permission from ref (1). Copyright 2022 The Authors. Published by American Chemical Society.

A GDE, instead of a conventional RRDE, enabled oxygen to be supplied directly to the back of the electrode without interference from the electrolyte, thus eliminating oxygen concentration gradients on the measured catalytic activity (Figure 1). The authors observed current densities up to −103 mA cm^–2^ at −0.36 V for Ni_3_(HITP)_2_, about 310 times greater than with a conventional RRDE that measured a current density of −0.6 mA cm^–2^.^1^ This marked improvement suggests that the catalytic performance of conductive MOFs toward ORR is greatly influenced by mass transport efficiency of O_2_, thus implying the importance of the careful design of a device. Control experiments under inert conditions confirmed that the current measured with the GDE was exclusively from the ORR.

Furthermore, the authors noted that MOFs with greater crystallinity tend to exhibit greater intrinsic surface area and electrical conductivity on top of the relationship between mass transport and ORR efficiency. Electrical conductivity and accessible surface area are critical components for the electrochemical surface area (ECSA) that contributes to the material’s catalytic performance. Using GDEs, the authors probed the influence of metal ion identity and mass loading on ORR performance. The authors found that Ni_3_(HITP)_2_ was a more effective ORR catalyst than Co_3_(HITP)_2_ or Cu_3_(HITP)_2_ due to its higher ECSA arising from higher crystallinity providing a higher density of active sites and greater electrical conductivity. Meanwhile, the mass loading studies revealed that, at high mass loading, mass activity diminishes, as much of the ECSA is not participating in the ORR.^1^

The critical importance of ECSA to ORR activity led the authors to explore a nanoscale technique, high-resolution scanning electrochemical cell microscopy (SECCM), that is not commonly used to study MOFs. This technique measures ORR current density while eliminating the effects of the mass transport through the bulk material by measuring the ORR in a single 50 nm diameter droplet of electrolyte on the surface of the electrode.^9^ At this scale, the geometric current densities can be effectively mapped on a micrometer scale. The ORR current density of Ni_3_(HITP)_2_ measured −1273 mA cm^–2^, 38 times greater than that measured with GDE, and a much larger mass activity. They further probed the intrinsic activity of Ni_3_(HITP)_2_ by adding 10 wt % PTFE to the Ni_3_(HITP)_2_ electrode ink to increase hydrophobicity and limit the ability of the aqueous electrolyte to impede oxygen diffusion. The enhanced oxygen diffusion led to a 67 mA cm^–2^ improvement compared to their previous GDEs, further demonstrating the crucial role of mass transport for an effective ORR.

This work by Dincă, Unwin, and co-workers showcases the critical distinction between extrinsic and intrinsic factors when evaluating the catalytic activity of porous materials. They have shown that previous measurements of the ORR activity of Ni_3_(HITP)_2_ were not a natural shortcoming of the material but of mass transport. By unlocking the inherent activity of Ni_3_(HITP)_2_ through improved mass transport, they have identified key parameters for future catalytic MOF materials. The presented methodology will foster the continued development of efficient conductive MOF electrocatalysts where their development may have otherwise been impeded by an underestimation of their catalytic abilities.
